# Ethnic Differences in Cardiac Rehabilitation Enrolment, Participation, and Mortality: A Retrospective Cohort Study from Auckland, New Zealand

**DOI:** 10.3390/jcm15166247

**Published:** 2026-08-12

**Authors:** Paul W. Marshall, Alana McCambridge, Sasha Douglas, Tia Kendal-Lindbom, Wendy Marshall, Michael O’Sullivan, Gayatri Ganesh, Cameron Walker, Nikki J. Earle, Stacey Reading, Jocelyne Benatar, Michael Kingsley

**Affiliations:** 1 School of Exercise, Sport and Rehabilitation Sciences, Faculty of Sciences, University of Auckland, Auckland 1142, New Zealandmichael.kingsley@auckland.ac.nz (M.K.); 2 Centre for Health and Rehabilitation Research, Faculty of Sciences, University of Auckland, Auckland 1142, New Zealand; 3 Person Centred Rehabilitation Research Centre, Faculty of Health and Environmental Sciences, Auckland University of Technology, Auckland 1142, New Zealand; 4 Greenlane Cardiovascular Services, Auckland City Hospital, Auckland 1142, New Zealand; 5 Department of Engineering Science and Biomedical Engineering, University of Auckland, Auckland 1142, New Zealand; 6 Te Pūnaha Matatini, Centre of Research Excellence, University of Auckland, Auckland 1142, New Zealand; 7 Department of Medicine, Faculty of Medical and Health Sciences, University of Auckland, Auckland 1142, New Zealand; 8 Holsworth Research Initiative, La Trobe Rural Health School, La Trobe University, Melbourne, VIC 3552, Australia

**Keywords:** cardiovascular disease, Māori, Pacific peoples, equity, participation

## Abstract

**Background/Objectives**: Ethnic inequities in cardiovascular disease and healthcare access are well recognised worldwide, but it is unclear whether these inequities translate into differences in cardiac rehabilitation participation and subsequent clinical outcomes. This study examined ethnic differences in cardiac rehabilitation enrolment, participation, and mortality outcomes within a large multi-ethnic cohort to determine whether the association between rehabilitation exposure and mortality differed across ethnic groups. **Methods**: This retrospective cohort study included 4940 consecutive patients referred to the Auckland cardiac rehabilitation programme between 2016 and 2023. Baseline characteristics, rehabilitation participation, and all-cause mortality were examined across ethnic groups. Associations with mortality were assessed using multivariable Cox proportional hazards models adjusted for demographic and clinical risk factors. **Results**: During a median follow-up of 5.6 years, 565 deaths occurred. Cardiac rehabilitation participation differed across ethnic groups (*p* < 0.001), with lowest participation among Pacific patients (44.3%) and highest participation among Asian patients (58.6%). Among attenders, total session attendance was similar across ethnic groups (*p* = 0.35). After adjustment, mortality risk was lower among Asian patients (HR 0.58, 95% CI 0.46–0.74, *p* < 0.001), but did not differ for Māori (HR 1.21, 95% CI 0.86–1.71) or Pacific patients (HR 1.03, 95% CI 0.79–1.36) compared with Europeans. Greater rehabilitation exposure was associated with lower mortality (HR 0.98 per session, 95% CI 0.97–0.99, *p* = 0.002), and no interaction between ethnicity and rehabilitation exposure was observed. **Conclusions**: Ethnic differences in cardiac rehabilitation were evident at the level of enrolment, but not in session exposure among attenders or in the association between rehabilitation participation and mortality. This study highlights the importance of distinguishing inequities in access and engagement from the effectiveness of rehabilitation once delivered.

## 1. Introduction

Cardiovascular disease remains a leading cause of mortality and morbidity in New Zealand [1], with Māori and Pacific peoples experiencing higher rates of cardiovascular disease, earlier disease onset, and poorer cardiovascular outcomes compared with European populations [2,3]. These disparities reflect a complex interaction of socioeconomic conditions, structural determinants of health, differential access to preventive services, and variation in the delivery and uptake of healthcare [4]. Cardiac rehabilitation is a core component of secondary prevention following acute cardiac events and includes structured exercise, education, and behavioural support aimed at reducing recurrent cardiovascular events and mortality [5,6,7]. However, participation in cardiac rehabilitation remains suboptimal in many health systems [8,9], with barriers to engagement including socioeconomic and logistical constraints, competing social responsibilities, health literacy, and aspects of service delivery [10,11,12]. While ethnic inequities in cardiovascular disease and healthcare access are well recognised in New Zealand and worldwide, it remains unclear whether these inequities translate into differences in cardiac rehabilitation participation and subsequent clinical outcomes.

Evidence from primary care suggests that inequities occur across multiple stages of the cardiovascular care pathway [2,3,10]. In New Zealand lower rates of cardiovascular risk assessment and secondary prevention medication use have been reported among Māori and Pacific peoples compared with other groups, alongside differences in access to revascularisation procedures [4]. Qualitative research examining Māori and Pacific families’ experiences of cardiovascular care has also identified barriers related to access, relationships with healthcare providers, cultural safety, and competing family and community responsibilities [13]. Together, these findings indicate that inequities may arise from both structural determinants of health and interactions with healthcare services, including cardiac rehabilitation.

Auckland has a highly diverse population that includes Māori, Pacific, and Asian communities. Lower participation in cardiac rehabilitation among Māori and Pacific patients has been reported [9,14], reflecting similar patterns observed across other outpatient and secondary prevention services. These participation differences raise concerns regarding whether inequities in access to, or engagement with, rehabilitation programmes may further exacerbate disparities in cardiovascular outcomes between ethnic groups. However, most studies have focused on referral or attendance patterns [9,10,13,14], with fewer examining whether differences in participation translate into differences in clinical outcomes once patients engage with rehabilitation. As differences in outcomes may arise from multiple points along the care pathway, including disease burden, age at presentation, acute treatment, and engagement with secondary prevention services, evaluating outcomes within cardiac rehabilitation cohorts may help determine whether observed disparities persist within the service itself or primarily reflect earlier differences in disease burden and healthcare access. More broadly, distinguishing inequities in participation from differences in rehabilitation effectiveness has implications beyond New Zealand, as lower utilisation does not necessarily imply reduced benefit once patients engage with rehabilitation.

In the New Zealand context, this work was informed by Te Tiriti o Waitangi, and specifically the principle of equity (oritetanga), which is a major goal of the New Zealand Health Strategy. The aim of this study was to examine ethnic differences in cardiac rehabilitation participation, rehabilitation attendance, and mortality outcomes within a large clinical cohort of patients referred to the Auckland cardiac rehabilitation programme. Specifically, the study examined whether participation and attendance differed across ethnic groups, whether these differences were associated with mortality outcomes after adjustment for demographic and clinical risk factors, and whether the association between cardiac rehabilitation participation and mortality, including the response relationship with session attendance, differed across ethnic groups. It was hypothesised that while differences between ethnic groups would be observed in presenting comorbidities and cardiac rehabilitation engagement, the association between rehabilitation participation and mortality would not differ between ethnic groups.

## 2. Methods

This was a retrospective cohort study that used routinely collected clinical data from the Auckland Health District outpatient cardiac rehabilitation service, linked to administrative health datasets. The study population comprised 4940 consecutive adult patients referred to the Auckland cardiac rehabilitation programme between 2016 and 2023 (Table 1). All patients attending the initial outpatient cardiac clinic following hospital discharge were included to allow evaluation of both cardiac rehabilitation participation and subsequent outcomes. Time zero was defined as the date of the initial outpatient clinic assessment. The study was approved by the Auckland Health Research and Ethics Committee (AHREC; AH22608) and the Auckland Hospital Research Committee (A9621). Given the retrospective design and use of routinely collected data, individual informed consent was not required.

### 2.1. Outpatient Clinics and Cardiac Rehabilitation Programme

The Auckland health district provides publicly funded outpatient cardiac services with no user fees or out-of-pocket expenses. Patients are routinely enrolled into outpatient follow-up following inpatient care at Auckland Hospital, with additional referrals from elective and private care pathways. Initial nurse specialist-led outpatient clinics, with medical oversight, occur within 1–3 weeks post-discharge and serve as the primary triage point for ongoing management and referral to cardiac rehabilitation. During these clinics, patients undergo medication review and optimisation, receive early guidance on secondary prevention, and are informed about available cardiac rehabilitation options [15,16].

Cardiac rehabilitation programmes were delivered in accordance with national guidelines [17] and comprised both lifestyle education and supervised exercise components. Detailed descriptions of patient assessment, exercise testing, exercise prescription, progression protocols, and programme delivery have been published previously [9,18]. As the present study focused on participation, ethnic differences, and long-term outcomes, only a brief overview of the rehabilitation programme is provided here. The lifestyle rehabilitation programme consisted of up to eight group-based education sessions delivered by nursing staff and multidisciplinary team members, addressing cardiovascular risk factors, medication adherence, nutrition, physical activity, and long-term self-management [9]. The exercise-based rehabilitation programme was delivered twice weekly over approximately eight weeks and was supervised by physiotherapists and nurse-specialists [18]. Sessions included aerobic exercise (e.g., treadmill walking and cycling) and resistance-based activities, with workloads individualised based on baseline clinical assessment.

Standard programme components were supplemented with service-level supports aimed at facilitating engagement and reducing barriers to participation. These included interpreter services across clinic consultations and rehabilitation sessions, language-specific education sessions (e.g., interpreter-supported group education for Mandarin-speaking patients), and prioritised wait-list access to exercise rehabilitation for Māori and Pacific patients to reduce risk of non-engagement. Additional practical supports included parking vouchers and transport assistance (taxi services and fuel vouchers), with transport support prioritised for patients identified as having financial or access barriers, including all Māori and Pacific patients.

### 2.2. Clinical Outcomes and Covariates

The primary outcome was all-cause mortality. Mortality events were identified through linkage of rehabilitation records to administrative datasets using the National Health Index (NHI) identifier.

### 2.3. Baseline Covariates

Baseline covariates included age, sex, ethnicity, primary cardiac diagnosis, procedure during index admission, and documented comorbidities, including tobacco use, heart failure, kidney disease, diabetes, and hypertension. Ethnicity was extracted from administrative records using standard national ethnicity classifications.

### 2.4. Ethnicity

Ethnicity was grouped into five categories: European, Māori, Pacific, Asian, and Other. The European category included New Zealand European, European, and Other European. The Asian category included Chinese, Indian, Southeast Asian, and Other Asian. The Pacific category comprised all individuals classified as Pacific Peoples within the source data, which included people of Pacific Island descent (e.g., Samoan, Tongan, Cook Islands Māori, Niuean, and other Pacific groups) aggregated within this classification. Māori was retained as a standalone category. All remaining ethnicities (including Middle Eastern, African, Latin American, and other unspecified groups) were classified as Other. Where multiple ethnicities were recorded, the primary recorded ethnicity was used for analysis. Multiple ethnicity entries were uncommon and did not materially influence group classification. The Other category was retained in all statistical analyses but not interpreted due to small sample size and heterogeneity.

### 2.5. Statistical Analysis

All analyses were conducted using IBM SPSS (version 27). Statistical significance was set at *p* ≤ 0.05.

Descriptive statistics were used to summarise baseline characteristics, cardiac rehabilitation participation, and session attendance across ethnic groups. Differences between ethnic groups were assessed using one-way analysis of variance (ANOVA) for continuous variables and chi-squared tests for categorical variables. Given the large sample size and balanced distribution across major ethnic groups, one-way ANOVA was considered sufficiently robust to moderate violations of normality assumptions. Post-hoc comparisons with Bonferroni’s correction were performed for continuous variables where overall differences were observed, to aid interpretation of group differences. No post-hoc analyses were undertaken for categorical variables.

Associations between ethnicity and mortality were examined using Cox proportional hazards regression, with European used as the reference group. Multivariable models were constructed in a staged approach. Candidate covariates were selected a priori for initial modelling based on clinical relevance, established predictors or mortality following cardiac events, and prior analyses of this cohort (age, gender, comorbidities). The final model retained age, smoking status, diabetes, hypertension, heart failure, and kidney disease because these variables contributed meaningfully to model performance. Subsequent models included cardiac rehabilitation participation and session attendance to assess whether rehabilitation engagement influenced observed associations. Cardiac rehabilitation participation was examined as a binary exposure based on attendance at one or more rehabilitation sessions following outpatient assessment. Timing data required for formal time-dependent exposure modelling were incomplete, and immortal time bias cannot be excluded. Because rehabilitation exposure occurred after outpatient assessment, sensitivity analyses excluding patients who died within 30 and 60 days of outpatient assessment were undertaken to assess the potential influence of early events, although these analyses do not eliminate immortal time bias.

Cardiac rehabilitation session attendance, including both lifestyle and exercise sessions, was examined as a continuous variable. To examine whether the association between cardiac rehabilitation and mortality differed across ethnic groups, interaction terms between ethnicity and session attendance (Ethnicity × Session) were included in the Cox regression models. For interaction analyses, session attendance was mean-centred to aid interpretation of model coefficients. As a sensitivity analysis, cardiac rehabilitation participation (binary variable) and total session attendance (continuous) were included simultaneously in the Cox regression models to distinguish the effects of rehabilitation initiation and accumulated exposure. Interaction terms between ethnicity and total session attendance were included to assess whether the dose–mortality relationship differed across ethnic groups under this specification. Additional sensitivity analyses adjusting for primary diagnosis and procedure were also performed to assess the robustness of observed associations. Hazard ratios (HRs) with 95% confidence intervals (CIs) were reported. Proportional hazards assumptions were assessed using Schoenfeld residuals.

## 3. Results

The European category (Table 1) comprised predominantly New Zealand European patients (n = 2198, 44.5%), the Asian category primarily Indian (n = 654, 13.2%) and Chinese patients (n = 347, 7.0%), and the Pacific category primarily Samoan (n = 215, 4.4%), Tongan (n = 147, 3.0%), and Cook Islands Māori patients (n = 104, 2.1%).

Baseline characteristics differed across ethnic groups (Table 1). Māori and Pacific patients were younger at presentation than European patients (mean age 60.8 ± 10.9 and 60.0 ± 11.4 years respectively vs. 68.0 ± 10.8 years, *p* < 0.001), with higher prevalence of cardiometabolic comorbidities, including diabetes, hypertension, and kidney disease, compared with Europeans (*p* < 0.001). Current smoking rates were highest among Māori and Pacific patients, while historical smoking was more common among Asian patients (*p* < 0.001). Differences in the distribution of primary diagnoses were observed across ethnic groups, with variation in selected diagnostic categories including stable angina, acute coronary syndrome of undefined type, and valve-related disease (*p* < 0.001). Differences in procedure were also observed, with variation in revascularisation and valve-related procedures across ethnic groups (*p* < 0.01), while other treatment categories did not differ.

Cardiac rehabilitation participation differed across ethnic groups (Table 2; *p* < 0.001), with lowest participation among Pacific patients (44.3%) and highest participation among Asian patients (58.6%). Among those who engaged with rehabilitation, total session attendance was similar across ethnic groups (mean 10.6 ± 7.7 sessions; *p* = 0.345). Lifestyle rehabilitation participation differed by ethnicity (*p* < 0.001) with lower numbers of Pacific (21.4%) and Māori patients (30.0%) engaging. Amongst those who participated, the number of sessions attended was lowest among Asian patients (4.6 ± 2.6, *p* < 0.001). Exercise rehabilitation participation was higher among Asian patients (40.9%; *p* < 0.001) although the number of exercise sessions attended was comparable between ethnicities (11.3 ± 6.4 sessions; *p* = 0.073).

**Figure 1:**
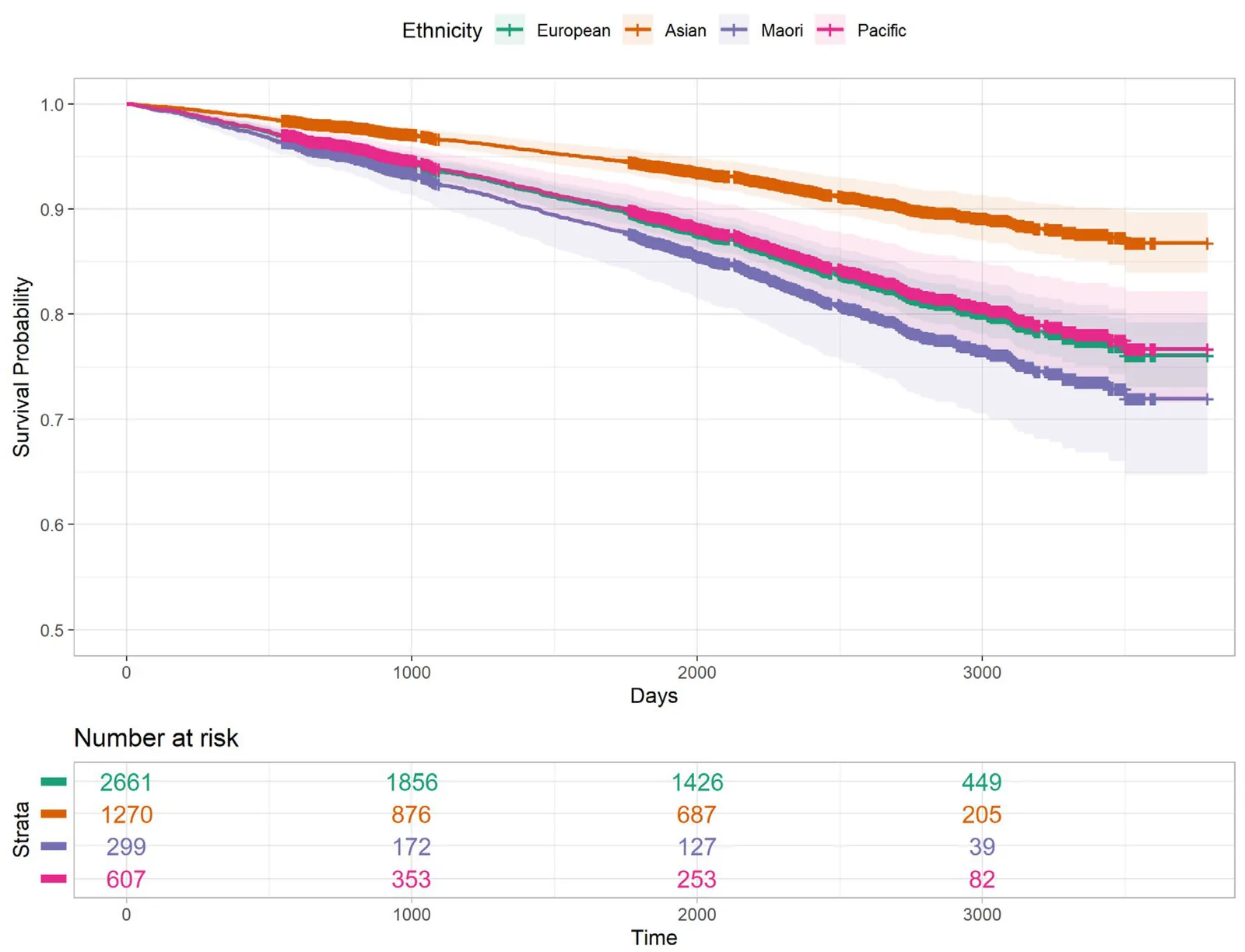
Kaplan–Meier survival curves for all-cause mortality stratified by ethnicity within the Auckland cardiac rehabilitation cohort. Shaded regions represent 95% confidence intervals. Numbers at risk for each ethnic group are displayed beneath the figure. Survival distributions differed across ethnic groups (log-rank *p* < 0.001).

There were 565 mortality events (11.4%) recorded within the cohort during follow-up (median follow-up 5.6 years: IQR 2.4 to 7.5 years). Unadjusted mortality differed across ethnic groups (Table 2; *p* < 0.001), with lower mortality observed among Asian patients (7.1%). Kaplan–Meier survival curves also differed across ethnic groups (log-rank *p* < 0.001; Figure 1), with Asian patients demonstrating higher survival probabilities over the follow-up period. In multivariable adjusted Cox regression analysis (Table 3), Asian ethnicity was associated with lower mortality compared with European patients (HR 0.58, 95% CI 0.46–0.74, *p* < 0.001). Mortality risk did not differ from European patients for Māori (HR 1.21, 95% CI 0.86–1.71) or Pacific patients (HR 1.03, 95% CI 0.79–1.36). Age, diabetes, heart failure, and kidney disease were independently associated with higher mortality risk (Table 3; *p* < 0.01).

Addition of cardiac rehabilitation participation as a binary exposure did not alter associations between ethnicity and mortality (Table 3). Rehabilitation participation was associated with a non-significant reduction in mortality risk (HR 0.85, 95% CI 0.72–1.01, *p* = 0.061). When total session attendance was included, greater cardiac rehabilitation exposure was associated with lower mortality risk (HR 0.98 per session, 95% CI 0.97–0.99, *p* = 0.002), while associations between ethnicity and mortality remained unchanged. The model-implied cumulative HR for 10 additional sessions was 0.83, corresponding to an approximate 17% lower mortality risk. Inclusion of interaction terms between ethnicity and cardiac rehabilitation session attendance did not demonstrate effect modification (*p* > 0.05). The inverse association between rehabilitation dose and mortality was consistent across ethnic groups. In exploratory analyses examining rehabilitation components separately, both lifestyle (HR 0.95 per session, 95% CI 0.93–0.98, *p* = 0.002) and exercise session attendance (HR 0.98 per session, 95% CI 0.97–0.99, *p* = 0.007) were associated with lower mortality risk, but inclusion of either component did not alter associations between ethnicity and mortality.

Sensitivity analyses excluding patients who died within 30 (n = 4) or 60 days (n = 11) of outpatient assessment did not alter associations between rehabilitation exposure, ethnicity, and mortality. Additional analyses undertaken to separate the effects of rehabilitation initiation from accumulated exposure showed no evidence of interaction between ethnicity and session attendance (all interaction *p* > 0.05), indicating that the session–mortality association remained consistent across ethnic groups. Additional adjustment for primary diagnosis or procedure did not alter the associations between ethnicity, rehabilitation exposure, and mortality outcomes, with all primary effect estimates remaining substantively unchanged.

## 4. Discussion

This study was designed to address an important gap in the evaluation of equity within cardiac rehabilitation by examining both engagement with rehabilitation and mortality outcomes within a large multi-ethnic Auckland cohort. Greater rehabilitation exposure was associated with lower mortality over a median follow-up of 5.6 years, and this relationship was consistent across ethnic groups, supporting the importance of rehabilitation engagement across the population. Lower rehabilitation enrolment among Māori and Pacific patients was evident, whereas session attendance and the association between rehabilitation exposure and mortality were consistent across ethnic groups. These findings indicate that inequities in cardiac rehabilitation are observable at the level of access and initial engagement, rather than differences in the association between rehabilitation participation and outcomes.

Much of the contemporary literature describes ethnic disparities in cardiac rehabilitation as a single construct, without distinguishing between access, participation, and outcomes [19,20,21]. The findings of the present study suggest that these domains should be considered separately. While differences in participation were observed, session exposure among attenders and the association between rehabilitation sessions and mortality were similar across ethnic groups. This indicates that disparities in utilisation should not be interpreted as evidence that the association between cardiac rehabilitation exposure and mortality differs across ethnic groups. These findings support a conceptual distinction between inequities in access to rehabilitation and differences in the observed association between rehabilitation exposure and outcomes. Although upstream factors influence who initiates and engages with programmes [19,20], the benefits associated with rehabilitation exposure appear comparable across ethnic groups among patients who participated.

Differences in cardiac rehabilitation participation by ethnicity are well described in the literature, including in New Zealand [8,13], and are commonly attributed to a range of social and structural determinants of health, including financial constraints, transport barriers, access to services, and broader healthcare system factors [4,19]. Importantly, disparities in participation persist even after accounting for socioeconomic factors such as income, indicating that structural and social determinants of health play a substantial role in shaping engagement with rehabilitation services [10]. Interventions targeting these barriers, including interpreter services, transport support, and culturally tailored programmes, have been proposed to improve engagement [20,21]. The Auckland cardiac rehabilitation service incorporated several of these approaches, including interpreter services, prioritised wait-list access, language-specific education sessions, behavioural strategies [22], and transport assistance targeted to patients with identified access barriers. Despite these measures, differences in participation persisted across ethnic groups, suggesting that barriers to engagement may extend beyond programme-level factors and reflect broader structural and socioeconomic influences on access to care. Māori and Pacific patients within this cohort were also substantially younger at presentation than European patients, with mean ages approximating 60 years. This likely reflects additional occupational, financial, and family responsibilities occurring during working age, which may further influence the ability to engage with outpatient rehabilitation programmes delivered during standard working hours. Together, these findings indicate that improving equity in cardiac rehabilitation requires greater emphasis on access and early engagement, as barriers to participation appear to extend beyond programme-level strategies.

Baseline differences in age, comorbidity burden, and clinical presentation were evident across ethnic groups. These factors are known to influence treatment selection and outcomes and reflect upstream determinants of health and care pathways that were not the focus of the present analysis. For example diabetes and current smoking were independently associated with mortality, highlighting their importance as modifiable risk factors within secondary prevention. While changes in these factors were not assessed, their distribution across ethnic groups suggests that targeted risk factor management may represent an important avenue for improving outcomes. Addressing these factors within and alongside cardiac rehabilitation may be particularly relevant for Māori and Pacific patients, given the higher burden of cardiometabolic risk observed at baseline.

However, after adjustment for age and comorbidities, mortality did not differ between Māori, Pacific, and European patients, while Asian patients demonstrated lower risk. These findings suggest that, within this cohort and after accounting for baseline differences, outcomes were comparable across the main ethnic groups of interest. This pattern is consistent with findings from the Multi-Ethnic New Zealand Study of Acute Coronary Syndromes (MENZACS) programme, where apparent ethnic differences in outcomes following acute coronary syndrome are attenuated after adjustment for overall clinical risk and are not fully explained by individual biological markers [23,24]. Māori patients have been shown to exhibit lower lipoprotein(a) concentrations despite higher rates of adverse outcomes, while associations between Māori ethnicity and clinical outcomes are no longer significant after accounting for a comprehensive clinical risk profile [23,24]. Together, these findings indicate that differences in outcomes are largely explained by underlying risk and broader determinants of health rather than ethnicity itself or isolated biological factors.

Although differences in participation between ethnic groups remain important, approximately half of all eligible patients did not attend any cardiac rehabilitation, indicative of the persistent worldwide problem of suboptimal enrolment in outpatient services. Thus barriers to participation extend beyond ethnicity-specific factors and highlight the need to better understand common determinants of enrolment across all patient groups. Given the consistent association between rehabilitation exposure and lower mortality across ethnic groups, improving overall enrolment and participation may represent an important opportunity to enhance outcomes, while continued efforts to address inequities experienced by Māori and Pacific patients remain warranted.

This study has several limitations. As an observational analysis, causal inference cannot be established, and residual confounding from unmeasured variables remains possible despite adjustment for key clinical factors. The analysis was conducted within a referred cohort, and findings may be influenced by selection into cardiac rehabilitation. Ethnicity was coded as a single category using the classification approach available within the dataset, which does not capture multi-ethnic identity and may obscure heterogeneity within groups. Important social and structural determinants of health, including socioeconomic status, deprivation, health literacy, and access-related factors, were not available and could not be accounted for. Data on medication use and adherence were also not available. In addition, detailed physiological and imaging measures were unavailable. Ethnic differences in cardiovascular physiology have been reported in some populations [25], and residual confounding from unmeasured physiological characteristics therefore cannot be excluded. Although formal time-dependent exposure modelling was not possible, early mortality before rehabilitation exposure was uncommon, and sensitivity analyses excluding early deaths did not significantly alter the findings. Because reliable dates of rehabilitation initiation were not available consistently, participation and accumulated session attendance could not be modelled as time-dependent exposures. Immortal time bias therefore cannot be excluded, and exclusion of deaths within 30 or 60 days only assessed the sensitivity of the findings to early events rather than removing this bias. Healthy-user bias may also have contributed, as patients who attended rehabilitation may have differed from non-attenders in unmeasured factors influencing both rehabilitation participation and long-term outcomes. Longer-term behaviour following programme completion was not captured, limiting insight into how sustained engagement may contribute to observed associations with long-term outcomes. Finally, quantitative data cannot capture the lived experience of rehabilitation therefore qualitative research is needed to understand what Māori and Pacific peoples experience within these services, what matters to them, and what barriers they face.

## 5. Conclusions

Distinguishing differences in enrolment, participation, and outcomes from cardiac rehabilitation is important in New Zealand, where Māori and Pacific Peoples experience a disproportionate burden of cardiovascular disease, and more broadly where differences in rehabilitation access and participation are often conflated with the benefits achieved once patients engage with rehabilitation. Māori and Pacific patients were younger, presented with a higher comorbidity burden, and had lower enrolment in rehabilitation than Europeans. No differences between ethnic groups were observed in session exposure among attenders or in the association between rehabilitation attendance and reduced mortality. These findings emphasise the importance of distinguishing inequities in access to rehabilitation from the effectiveness of rehabilitation once delivered and support a focus on improving participation and early engagement for Māori and Pacific Peoples as key targets for reducing inequities in cardiovascular outcomes.

## Figures and Tables

**Table 1:** Baseline demographics, comorbidities, primary diagnosis, procedure, and cardiac rehabilitation participation for the total cohort stratified by ethnicity (European, Asian, Pacific Peoples, Māori, Other), Auckland cardiac rehabilitation programme, 2016–2023. * is *p* < 0.05 and ** is *p* < 0.01 from other groups.

	Overall (*n* = 4940)	European (*n* = 2661)	Asian (*n* = 1270)	Pacific Peoples (*n* = 607)	Māori (*n* = 299)	Other (*n* = 103)	Main Effect, Ethnicity
Mean age ± SD, years	65.0 ± 11.7	68.0 ± 10.8 **	62.6 ± 12.1	60.0 ± 11.4	60.8 ± 10.9	59.2 ± 10.7	*p* < 0.001
Women, *n* (%)	1396 (28.3)	765 (28.7)	371 (29.2)	149 (24.5)	83 (27.8)	28 (27.2)	*p* = 0.27
Hospital admissions year prior (mean ± SD)	2.0 ± 1.8	2.0 ± 1.7	1.8 ±1.5	2.1 ±1.9	2.5 ± 3.5 *	2.4 ± 2.1 *	*p* < 0.001
Comorbid Conditions, *n* (%)							
Historical smoker	2147 (43.5)	1290 (48.5)	851 (67)	261 (43.0)	138 (46.2)	39 (37.9)	*p* < 0.001
Current smoker	800 (16.2)	333 (12.5)	152 (12.0)	174 (28.7)	114 (38.1)	27 (26.2)	*p* < 0.001
Diabetes	1514 (30.6)	474 (17.8)	564 (44.4)	321 (52.9)	115 (38.5)	40 (38.8)	*p* < 0.001
Heart failure	550 (11.1)	277 (10.4)	102 (8.0)	96 (15.8)	65 (21.7)	10 (9.7)	*p* < 0.001
Kidney disease	659 (13.3)	294 (11.0)	168 (13.2)	138 (22.7)	45 (15.1)	14 (13.6)	*p* < 0.001
Hypertension	3310 (67)	1662 (62.5)	868 (68.3)	490 (80.7)	223 (74.6)	67 (65)	*p* < 0.001
Primary diagnosis, *n* (%)							
NSTEMI	1407 (28.5)	754 (28.3)	369 (29.1)	179 (29.5)	75 (25.1)	30 (29.1)	*p* = 0.69
STEMI	788 (16.0)	419 (15.7)	204 (16.1)	105 (17.3)	45 (15.1)	15 (14.6)	*p* = 0.90
Stable Angina	772 (15.6)	393 (14.8)	241 (19.0)	80 (13.2)	35 (11.7)	23 (22.3)	*p* < 0.001
Unstable Angina	390 (7.9)	213 (8.0)	98 (7.7)	44 (7.2)	24 (8.0)	11 (10.7)	*p* = 0.80
Atrial Fibrillation/Flutter	50 (1.0)	31 (1.2)	8 (0.6)	6 (1.0)	5 (1.7)	0 (0)	*p* = 0.46
Valve Disease	378 (7.6)	271 (10.2)	39 (3.1)	46 (7.6)	19 (6.4)	3 (2.9)	*p* < 0.001
ACS Undefined	655 (13.3)	307 (11.5)	211 (16.6)	79 (13.0)	44 (14.7)	14 (13.6)	*p* < 0.001
Cardiomyopathy	111 (2.2)	72 (2.7)	12 (0.9)	13 (2.1)	12 (4.0)	2 (1.9)	*p* = 0.018
Heart Failure	52 (1.1)	22 (0.8)	15 (1.2)	7 (1.2)	6 (2.0)	2 (1.9)	*p* = 0.13
Other	337 (6.8)	179 (6.7)	73 (5.7)	48 (7.9)	34 (11.4)	3 (2.9)	*p* = 0.044
Primary procedure, *n* (%)							
PCI	2123 (43.0)	1167 (43.9)	589 (46.4)	216 (35.6)	101 (33.8)	50 (48.5)	*p* < 0.001
Angiogram	1353 (27.4)	687 (25.8)	374 (29.4)	165 (27.2)	101 (33.8)	26 (25.2)	*p* = 0.005
CABG	635 (12.9)	313 (11.8)	165 (13.0)	102 (16.8)	41 (13.7)	14 (13.6)	*p* = 0.11
Valve	342 (6.9)	224 (8.4)	37 (2.9)	54 (8.9)	24 (8.0)	3 (2.9)	*p* < 0.001
Medical Management	88 (1.8)	37 (1.4)	22 (1.7)	18 (3.0)	9 (3.0)	2 (1.9)	*p* = 0.10
Other	399 (8.1)	233 (8.8)	83 (6.5)	52 (8.6)	23 (7.7)	8 (7.8)	*p* = 0.72

Abbreviations: NSTEMI, non-ST-segment elevated myocardial infarction; STEMI, ST-segment elevated myocardial infarction; ACS, acute coronary syndrome; PCI, percutaneous coronary intervention; CABG, coronary artery bypass graft. Post-hoc comparisons were performed and key differences are described in the text.

**Table 2:** Participation in cardiac rehabilitation, the number of lifestyle and exercise rehabilitation sessions attended by patients, and mortality events. * is *p* < 0.05 from all other groups.

	Overall (*n* = 4940)	European (*n* = 2661)	Asian (*n* = 1270)	Pacific Peoples (*n* = 607)	Māori (*n* = 299)	Other (*n* = 103)	Main Effect, Ethnicity
Participation							
Any cardiac rehabilitation session, n (%)	2590 (52.4)	1375 (51.7)	744 (58.6)	269 (44.3)	149 (49.8)	53 (51.5)	*p* < 0.001
Total sessions, mean ± SD	10.6 ± 7.7	10.6 ± 7.8	11.0 ±7.7	10.5 ± 8.2	9.6 ± 7.3	10.6 ± 7.8	*p* = 0.345
Overall Attendance (attenders only)							
Lifestyle attendance, n (%)	1814 (36.7)	1083 (40.7)	482 (37.9)	130 (21.4)	90 (30.0)	29 (28.2)	*p* < 0.001
Lifestyle sessions, mean ± SD	5.1 ± 2.7	5.4 ± 2.6 *	4.6 ± 2.6	4.9 ± 2.7	5.0 ± 3.1	5.2 ± 2.4	*p* < 0.001
Exercise attendance, n (%)	1626 (32.9)	763 (28.6)	520 (40.9)	204 (33.6)	99 (33.1)	40 (38.8)	*p* < 0.001
Exercise sessions, mean ± SD	11.3 ± 6.4	11.5 ± 6.2	11.5 ± 6.3	10.7 ± 7.3	9.9 ± 6.5	10.1 ± 6.3	*p* = 0.073
Mortality (n, %)	565 (11.4)	359 (13.5)	90 (7.1)	69 (11.4)	42 (14.0)	5 (4.9)	*p* < 0.001

**Table 3:** Multivariable Cox proportional hazards models examining associations between ethnicity, cardiac rehabilitation participation, and all-cause mortality. Hazard ratios (HRs) with 95% confidence intervals (CIs) are presented. Ethnicity is modelled using indicator coding with European patients as the reference group, such that all estimates represent relative risk compared with European patients.

	Model A	Model B	Model C
Ethnicity			
Asian	0.58 (0.46 to 0.74), *p* < 0.001	0.59 (0.46 to 0.75), *p* < 0.001	0.59 (0.46 to 0.75), *p* < 0.001
Pacific Peoples	1.03 (0.79 to 1.36), *p* = 0.81	1.02 (0.77 to 1.34), *p* = 0.89	1.01 (0.77 to 1.33), *p* = 0.94
Māori	1.21 (0.86 to 1.71), *p* = 0.27	1.23 (0.87 to 1.73), *p* = 0.24	1.22 (0.87 to 1.72), *p* = 0.25
Other	0.54 (0.22 to 1.30), *p* = 0.17	0.54 (0.22 to 1.32), *p* = 0.18	0.54 (0.22 to 1.33), *p* = 0.18
Comorbidities			
Age	1.08 (1.07 to 1.09), *p* < 0.001	1.08 (1.07 to 1.09), *p* < 0.001	1.08 (1.07 to 1.09), *p* < 0.001
Current smoker	1.53 (1.19 to 1.98), *p* < 0.001	1.49 (1.15 to 1.93), *p* = 0.002	1.46 (1.13 to 1.88), *p* = 0.004
Diabetes	1.68 (1.39 to 2.03), *p* < 0.001	1.67 (1.38 to 2.02), *p* < 0.001	1.66 (1.37 to 2.00), *p* < 0.001
Heart Failure	2.68 (2.21 to 3.24), *p* < 0.001	2.64 (2.18 to 3.19), *p* < 0.001	2.66 (2.20 to 3.22), *p* < 0.001
Kidney Disease	2.03 (1.67 to 2.46), *p* < 0.001	2.02 (1.66 to 2.45), *p* < 0.001	2.00 (1.65 to 2.43), *p* < 0.001
Hypertension	1.04 (0.85 to 1.28), *p* = 0.68	1.04 (0.85 to 1.28), *p* = 0.69	1.05 (0.86 to 1.29), *p* = 0.62
Rehabilitation	X	0.85 (0.72 to 1.01), *p* = 0.061	X
Total Sessions	X	X	0.98 (0.97 to 0.99), *p* = 0.002

Model A includes ethnicity and baseline covariates; Model B additionally includes cardiac rehabilitation participation (binary); Model C includes total rehabilitation session attendance (continuous).

## Data Availability

Data from this study are not publicly available, and no ethical approval has been received for release of data compiled from this study.
